# Nutritional Status Indices and Monoclonal Gammopathy of Undetermined Significance Risk in the Elderly Population: Findings from the National Health and Nutrition Examination Survey

**DOI:** 10.3390/nu15194210

**Published:** 2023-09-29

**Authors:** Linfeng Li, Mengrui Wu, Zhengyu Yu, Ting Niu

**Affiliations:** 1 Department of Hematology, West China Hospital, Sichuan University, Chengdu 610041, China; 2016141241194@stu.scu.edu.cn (L.L.); jokecook@126.com (Z.Y.); 2 Department of Obstetrics and Gynecology, Key Laboratory of Birth Defects and Related Diseases of Women and Children of MOE and State Key Laboratory of Biotherapy, West China Second Hospital, Sichuan University and Collaborative Innovation Center, Chengdu 610041, China; wumengruiscu@163.com

**Keywords:** MGUS, nutritional status, CONUT, PNI, plasma cell neoplasm, NHANES

## Abstract

Objective: Although several studies have found dietary intake is related to multiple myeloma (MM) and its precursor status risks, the role of one’s nutritional status has been ignored and its role in plasma cell neoplasm development is still unclear. This study aimed to explore the relationship between various clinical indices of nutritional status and the risk of monoclonal gammopathy of undetermined significance (MGUS) in the population. Methods: We selected 9520 participants from the NHANES III and NHANES 1999–2004 studies. Controlling nutritional status index (CONUT), prognostic nutritional index (PNI), geriatric nutritional risk index (GNRI) and body mass index (BMI) were calculated as indices of nutritional status of the participants. Associations between nutritional indices and MGUS were investigated using multiple logistic regression, subgroup analysis, and an RCS model. Results: In our study, 266 participants had MGUS, with a prevalence of 2.79%. This study found that CONUT and PNI identified populations with poor nutritional status and had a significant positive correlation with the risk of MGUS. In multivariate logistic regression, compared with the lower CONUT score (<3) group, the OR for the group with higher scores (≥3) was 1.805 (95%CI: 1.271, 2.564). Compared with the lowest quartile group, the highest quartile PNI score group had an OR of 0.509 (95%CI: 0.290, 0.896). GNRI had no significant correlation with the risk of MGUS, with an OR of 0.737 (95%CI: 0.443, 1.227). Conclusion: This study found that older adults with CONUT and PNI scores indicating poorer nutrition had a higher risk of MGUS.

## 1. Introduction

MGUS is a premalignant plasma cell dyscrasia. Among the inhabitants of western countries, the incidence rate in people over 50 years old is 3.2% [1,2]. A definitive analysis of 77,000 people enrolled in a previous prospective population-based cancer screening trial showed that multiple myeloma (MM) is always preceded by a precursor condition, which is monoclonal gammopathy of undetermined significance (MGUS), and 1% of MGUS patients progress to other more malignant plasma cell diseases such as multiple myeloma each year [3]. Current studies have found that the incidence risk of MGUS is significantly associated with age, gender, and race. In the United States, the incidence rate for women is 2.7%, while the rate for men is higher at 4.0% [4]. Importantly, compared with whites, African Americans have a two-fold or higher risk of developing multiple myeloma [5]. The higher frequency of multiple myeloma in blacks results from a higher incidence of the precursor abnormality MGUS, while the hazard of MGUS progressing to multiple myeloma is identical.

In most epidemiological studies of tumors, body mass index (BMI) as an index of nutritional status has been found to be associated with a higher risk of tumors in obese people, including MM [6,7,8]. At the same time, the risk of MGUS progression is higher in obese people [9]. However, few studies have explored the relationship between poor nutritional status and tumor incidence risk. Controlling nutritional status (CONUT), geriatric nutritional risk index (GNRI) and prognostic nutritional index (PNI) are indices used to assess the nutritional status of hospitalized patients and tumor patients [10,11,12]. Some studies have shown that these indices can be used as predictors of prognosis for heart disease [13,14], autoimmune diseases [15,16] and surgical patients [17]. However, these indices have only been used in people with some disease in the past, and few studies have explored if these indices have a correlation with tumor incidence in the general population.

Several studies have demonstrated a correlation between dietary intake and plasma cell neoplasm incidence. However, in nutritional studies, it is challenging to isolate the effects of single foods on health outcomes due to the varying combinations of foods consumed by individuals [18]. Therefore, examining overall dietary patterns and nutritional status can provide valuable insights into the links between nutrition and cancer [19].

In this study, in order to determine the relationship between nutritional status and plasma cell neoplasm, we explored for the first time, the correlations between the nutritional status indices and the incidence of MGUS, a precursor condition of MM, in the US population.

## 2. Methods

### 2.1. Study Design and Population

The National Health and Nutrition Examination Survey (NHANES) is a population-based cross-sectional survey. Data for our study were obtained from the NHANES III and NHANES 1999–2004. The survey is conducted by the National Center for Health Statistics and combines interviews and physical examinations to provide comprehensive data on demographics, socioeconomics, diet, health care, laboratory tests and health. A stratified multistage complex sampling plan is used to identify participants and select a sample that is nationally representative of the non-institutionalized US civilian population, with a higher sampling of older adults, non-Hispanic blacks, Mexican-Americans, and other groups. More detailed information on respondent recruitment, survey design and data collection can be obtained online [20,21].

### 2.2. Assessment of MGUS

The MGUS testing was performed at the Protein Immunology Laboratory of the Mayo Clinic in Rochester, Minnesota, USA, and the specific experimental techniques have been detailed in a previous study [1]. Serum protein agarose gel electrophoresis was performed for all subjects first. If samples with uncertain and definite M-proteins were found in electrophoresis, these samples were further tested for serum protein immunofixation electrophoresis and serum free light chain detection. The MGUS subtype was determined according to the results of immunofixation electrophoresis. All tests and interpretations were performed by personnel who did not know any demographic or other details of the tested samples.

### 2.3. Analytic Samples

Because the immunofixation electrophoresis and serum protein electrophoresis were accessible in NHANE III and NHANE 1999–2004, our study included a total of 33,197 participants and 31,126 participants from these two studies, respectively. We excluded participants who did not meet the following criteria: missing age data and those who were under 50 years old (*n* = 48,176), those who did not undergo MGUS testing (*n* = 3727), those who were missing nutritional status indices (*n* = 1975), and those who were missing covariates used in multivariate logistic regression (PIR, education, alcohol consumption, smoking status) (*n* = 935). Among all the participants, 9520 were included in the final analysis, of whom 266 met the diagnostic criteria for MGUS. The 9520 NHANES participants included in our study represented 49.87 million non-institutionalized elder residents of the United States.

### 2.4. Assessments of Nutritional Status

In this study, we used four nutritional indices to assess the nutritional status of the population: GNRI, PNI, CONUT, and BMI. The specific calculation methods of each index are shown in Table 1. GNRI and PNI were classified by quartiles, and participants in the first quartile (lowest score) indicated a relatively higher risk of malnutrition, while participants in the fourth quartile (highest score) indicated a relatively lower risk of malnutrition. The CONUT grouping was based on clinically meaningful cut-off value reported in previous studies [22,23,24,25], and the population was divided into low CONUT group (<3 scores) and high CONUT group (≥3 scores), with the high CONUT group having a relatively higher risk of malnutrition.

### 2.5. Covariates

Sociodemographic covariates included age, sex, race (non-Hispanic white, non-Hispanic black and other), education level divided into high school and above and below high school, and poverty income ratio (PIR) (≤1.35, 1.36–3, >3). The total daily energy intake measured by kcal was obtained from dietary data. Lifestyle-related covariates included smoking and drinking. Smoking status was defined by two questions (“Have you smoked at least 100 cigarettes in your lifetime?” and “Do you smoke now?”) and categorized into three groups (never smoked, former smoker, current smoker). Habitual drinking status was defined by one question (“Have you ever had 12 drinks of any kind of alcoholic beverage in any one year?”).

### 2.6. Statistical Analysis

All statistical analyses were performed in R (version 4.1.3). We used the Mobile Examination Center (MEC) exam weights provided by NHANES to account for the complex survey sampling design, the changes in design across survey cycles and the oversampling of specific subgroups. Continuous variables following a normal distribution were expressed as mean ± standard deviations and others were summarized using means and interquartile ranges (IQR). All the categorical variables were shown as percentages. For continuous variables, the *t*-test or Mann–Whitney test was applied, and chi-squared tests was applied for categorical variables. A 0.05 *p*-value was considered statistically significant. We used univariate and multivariate logistic regression models to examine the relationship between nutritional status indices and the likelihood of developing MGUS. In the multivariate logistic regression models, model 1 adjusted for age and sex, model 2 additionally adjusted for race, income and education level, and model 3 further adjusted for smoking and drinking status and total daily energy intake. To further explore the relationship between nutritional status indices and MGUS, we applied a restricted cubic spline (RCS) fitting model and performed a subgroup analysis. A two-sided *p* value of 0.05 was defined as statistically significant. Likelihood ratio tests were used to test for interaction between subgroups.

## 3. Results

### 3.1. Characteristics of the Study Population

**Figure 1:**
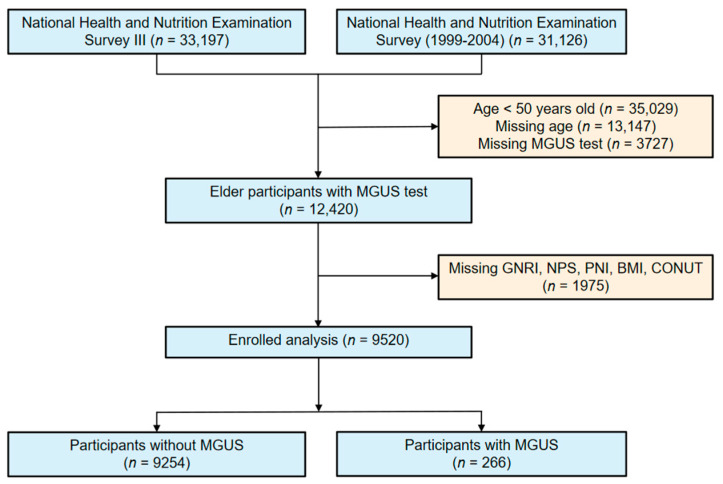
Flowchart for choosing participants from the National Health and Nutrition Examination Survey.

This study investigates the characteristics of participants with Monoclonal Gammopathy of Undetermined Significance (MGUS) among individuals enrolled in NHANES III and NHANES (1999–2004). A total of 9520 participants were included in this study (Figure 1), among whom 266 participants were confirmed to have MGUS through serum immunofixation, resulting in an overall prevalence rate of 2.8%. The baseline demographic and laboratory parameters of all participants are presented in Table 1. Among the participants diagnosed with MGUS, 48.29% were aged above 70 years, 53.56% were male, 11.04% were of African American ethnicity, and the median age was 68.83 years.

Comparative analysis revealed significant differences in demographic and baseline clinical characteristics between MGUS and non-MGUS participants. Participants with MGUS were found to be older (48.71% vs. 29.71%), more likely to be male (53.56% vs. 46.32%), and had a higher representation of Blacks (11.04% vs. 7.68%). Additionally, MGUS participants exhibited lower levels of education compared with those without MGUS. The prevalence of smoking and drinking was higher among MGUS participants. Furthermore, MGUS participants had significantly lower serum albumin levels (40.36 [39.62, 41.11] g/L) compared to non-MGUS participants (41.97 [41.75, 42.18] g/L), as well as significantly lower serum total cholesterol levels (208.65 [200.98, 216.32] mg/dL) compared with non-MGUS participants (216.84 [215.28, 218.40] mg/dL) (Table 2).

Notably, our results indicate that MGUS participants exhibited potentially poorer nutritional status with lower PNI scores (median [IQR], 50.50 [46.50, 53.75] vs. 52.00 [49.00, 55.50], *p* < 0.001), lower GNRI scores (median [IQR], 113.35 [104.88, 120.46] vs. 114.28 [107.85,121.91], *p* = 0.048).

### 3.2. Association of Nutritional Indices and Prevalence of MGUS

**Figure 2:**
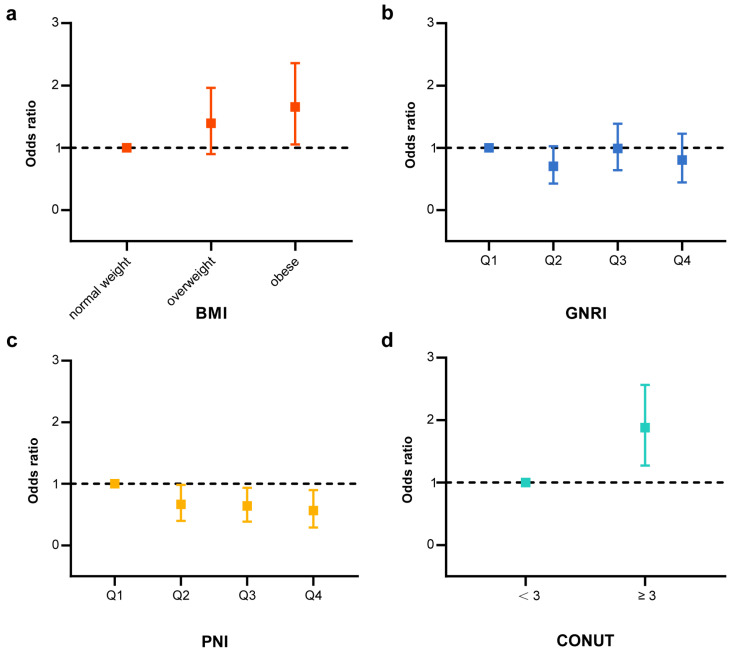
Association between the nutritional indices and MGUS (Model 3) adjusted for age, sex, ethnicity, income, education level, smoking status, drinking status and total daily energy intake. (**a**) Risk of MGUS by levels of BMI (Model 3) adjusted for age, sex, ethnicity, income, education level, smoking status, drinking status and total daily energy intake. (**b**) Risk of MGUS by quantile for GNRI (Model 3) adjusted for age, sex, ethnicity, income, education level, smoking status, drinking status and total daily energy intake. (**c**) Risk of MGUS by quantile for PNI (Model 3) adjusted for age, sex, ethnicity, income, education level, smoking status, drinking status and total daily energy intake. (**d**) Risk of MGUS by levels of CONUT (<3 or ≥3) adjusted for age, sex, ethnicity, income, education level, smoking status, drinking status and total daily energy intake.

Using established nutritional indices systems, including the CONUT assessment for “low risk” (<3) and “high risk” (≥3), BMI categorization into “normal weight”, “overweight”, and “obesity”, as well as quartiles for PNI and GNRI, we conducted a logistic regression analysis to estimate the odds ratios (ORs) for MGUS risk. After adjusting for age, gender, ethnicity, socioeconomic status, education, smoking history, and alcohol consumption, the multivariate logistic regression analysis revealed that being obese (OR = 1.553, 95% CI = 1.023–2.358; *p* = 0.039) and having higher CONUT values (OR = 1.805, 95% CI = 1.271–2.564; *p* = 0.002) were associated with an increased risk of MGUS. In contrast, participants in the highest quartile of PNI (OR = 0.509, 95% CI = 0.296–0.896; *p* = 0.020) and GNRI (OR = 0.737, 95% CI = 0.443–1.227; *p* = 0.233) showed a reduced risk of MGUS (Table 3, Figure 2).

In order to investigate the potential nonlinear relationship between nutritional indices and the incidence of MGUS, we conducted a Restricted Cubic Spline (RCS) analysis placing three knots (10%, 50%, 90%) according to Harrell’s recommended percentiles by using the R package ‘rms’ [26] and plotted the distribution of BMI, GNRI, PNI among all participants. (Supplementary Figure S1). The RCS analysis was performed as continuous variables. The results of the RCS analysis revealed a nonlinear negative correlation between GNRI and MGUS incidence (age and gender adjusted *p* for nonlinear = 0.038; multivariable adjusted *p* for nonlinear = 0.031). As GNRI decreased, the risk of MGUS increased rapidly, especially when GNRI was less than 113.634 (Supplementary Figure S1c,d). However, we did not observe any nonlinear associations between BMI and MGUS (nonlinear test, adjusted *p* for nonlinear association after accounting for age and gender = 0.229; adjusted *p* for nonlinear association after adjusting for multiple confounding factors = 0.245) or between PNI and MGUS (age and gender adjusted *p* for nonlinear = 0.084; multivariable adjusted *p* for nonlinear = 0.108).

### 3.3. Subgroup Analysis of MGUS Participants Based on Nutritional Indices

**Figure 3:**
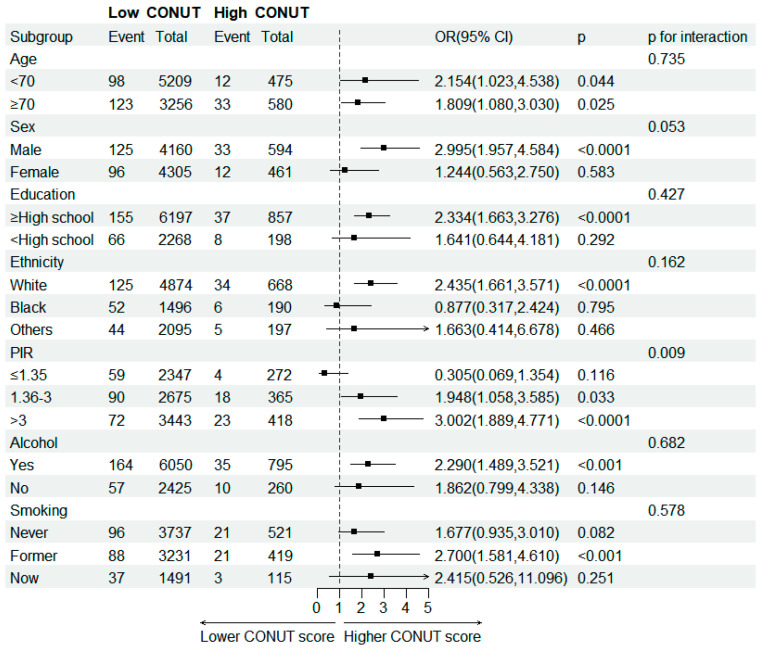
Subgroup analysis of association between CONUT score and MGUS stratified by age, sex, education level, ethnicity, PIR, smoking and drinking status.

This study conducted subgroup analyses of participants with MGUS based on four nutritional indices: CONUT, BMI, PNI, and GNRI. The relationship between these nutritional indices and MGUS incidence was explored in different subgroups of the study population stratified by covariates. The positive association between CONUT scores and incidence of MGUS was consistently observed across the stratified subgroups (Figure 3). The interaction between CONUT score and PIR in the incidence of MGUS was significant (*p* for interaction = 0.009). The association between CONUT score and risk of MGUS was more robust in the higher income population relative to the poverty threshold population (PIR > 3) than in the lower group. The interaction between BMI in the incidence of MGUS also showed a more significant association (*p* for interaction = 0.049) in the group formal smoker than in the group never smoked (Table S1). We observed an increased risk of MGUS associated with lower PNI score among older participants (*p* for interaction = 0.005) (Table S2). All covariates did not influence the relationship between GNRI and MGUS outcome (Table S3).

## 4. Discussion

Our study is the first to find a correlation between the nutritional status indices PNI and CONUT and the risk of MGUS. Relative poorer nutritional status assessed by PNI or CONUT is positively associated with the risk of MGUS. At the same time, we also found that obese people have a higher risk of MGUS, which is consistent with previous studies [27,28,29,30]. However, in our study, GNRI, as an index of nutritional status in the elderly, was not significantly correlated with the risk of MGUS.

PNI, CONUT and GNRI are all used in clinical practice to assess the nutritional status of patients. These indices have predictive effects on the prognosis of various types of tumors such as gastric cancer [31], pancreatic cancer [32], colorectal cancer [17], esophageal cancer [33], ovarian cancer [23] and lymphoma [34]. These nutritional status indices have never been used to assess the risk of tumor incidence, but the three indices all include serum albumin levels in the calculation of nutritional status, which can indicate malnutrition status, especially in patients after surgery and undergoing chemotherapy. The decrease in serum albumin level has been reported in some studies as a risk factor for cancer incidence [35]. One study examined the association between circulating liver function markers including albumin and colorectal cancer (CRC) risk in a large prospective cohort of 375,693 UK Biobank participants. The study followed up the participants for a median of 10 years and identified 2662 incident cases of CRC. This finding suggested higher levels of albumin were associated with a lower risk of CRC, and the HRs (95% CIs) for CRC comparing the highest to the lowest decile of albumin were 0.66 (0.55–0.79) [36]. Another study analyzed the association between albumin, bilirubin, and uric acid levels and cancer risk in a prospective population-based study of 25,540 volunteers from Germany in the age range between 35 and 65 years. The average follow-up duration in the cohort was 14.8 years with 627 breast cancer cases in women. Albumin levels were inversely associated with breast cancer risk, and the HRs (95% CIs) comparing the highest to the lowest quartile of albumin were 0.71 (0.51, 0.99) [37]. For MM, albumin level is an important factor for prognosis risk stratification after diagnosis [38,39,40]. Moreover, in a 34-year long-term follow-up study of MGUS at Mayo Clinic, it was found that only M protein level and albumin level were independent risk factors for IgM MGUS progression [41]. This implies that albumin level may play an important role in the incidence and progression of plasma cell neoplasm, but its mechanism is not clear. Previous studies have proposed various hypotheses for the increase in cancer risk caused by low albumin concentration, including malnutrition due to neoplasm proliferation [42], antioxidant properties [43] and inhibition of albumin synthesis due to systemic inflammation [44]. Past studies have found that albumin can act as an important extracellular antioxidant by scavenging reactive oxygen species and nitric oxide through its 34th thiol group, protecting cell membranes and DNA from damage [43]. In addition to reflecting a person’s nutritional status to some extent, low albumin levels may increase the risk of malignant tumors due to poor nutritional status, but this is only a speculation and there is no relevant epidemiological study.

However, GNRI score based on albumin level did not show a significant correlation with MGUS risk in our study. The possible reason is that the two nutritional status indices, PNI and CONUT, also take into account immune factors by including lymphocyte count in the calculation. Previous studies have suggested that immune status changes may vary with plasma cell neoplasm disease status [45]. A study investigated the count and proportion of lymphocytes in normal people, MGUS and MM patients, and found that the total lymphocyte count was significantly reduced in MGUS and MM patients [46]. Another study also found that the subgroups of lymphocytes changed, and the proportion of Treg cells in MGUS and MM populations was 25% and 26%, respectively, while the proportion of Treg cells in the normal population was 14% [47]. These results indicate that there is a certain degree of immune dysregulation in the MGUS population, which may cause a series of hematological malignancies. Of course, the immune dysregulation may also be caused by the interaction between tumor clone cells and the bone marrow microenvironment. Therefore, one reason PNI and CONUT were found to be correlated with MGUS risk in our study could be that they integrated both nutritional status and immune status considerations. Additionally, serum albumin, lymphocyte counts, and total cholesterol levels have a comparable impact on the CONUT score, whereas the PNI score is primarily determined by serum albumin levels and lymphocyte counts. Several studies have identified the cholesterol level as a nutritional parameter associated with the incidence of MM. One large cohort study comprising 116,728 individuals found an inverse association between the incidence of certain hematologic malignancies, including MM, and the levels of high-density lipoprotein cholesterol (HDL-C) and its crucial component, apolipoprotein A1 [48]. Another large cohort study in Korea similarly revealed that low baseline lipid levels (total cholesterol, HDL-C, and low-density lipoprotein cholesterol) were associated with a higher hazard ratio of MM incidence [49]. It is still unclear whether low cholesterol levels were a causal factor in MM or simply a coincidence. In our study, we also noted significantly lower serum total cholesterol levels in MGUS cases. And this might be one reason for the more significant association in CONUT compared with PNI.

In previous studies on MGUS epidemiology, the main findings were that the risk of disease increased with age, men had a higher incidence than women, and black people had a higher incidence [5]. In the previous two NHANES studies, similar results were also reported [4,50]. From the perspective of nutritional epidemiology, some studies have explored the relationship between dietary intake and MGUS or MM incidence. One meta-analysis mainly assessed the relationship between fish intake and MM risk. The article collected five case-control studies involving 1366 MM patients and 8259 controls and found that compared with the lowest intake, the highest intake of fish was significantly inversely associated with MM risk (relative risk 0.65, 95% confidence interval 0.46–0.91), but the results had high heterogeneity (I2 = 55.6%) [51]. Another cohort study in Iceland followed up the population for a long time. The article found that eating fruit at least three times a week during adolescence was associated with a lower incidence of MGUS, while eating fruit at least three times a week after MGUS diagnosis was associated with a lower risk of MM progression [52]. However, some studies have reached inconsistent conclusions. A case-control study in 2007 surveyed women in Connecticut, USA, and found that the intake of fruits, vegetables, meat, fish, main dishes, lunch foods, dairy products, sweets and beverages had no significant association with multiple myeloma risk [53]. Nevertheless, none of these studies assessed the nutritional status of the population to analyze whether it was related to MGUS incidence, and our exploration expands the existing evidence on the relationship between MGUS incidence and population nutritional status.

To our knowledge, this is the first study to offer insight into the influence of nutritional status on the risks of plasma cell neoplasms by analyzing the association between nutritional indices and MGUS incidence. Our study also has some limitations. First, our study is based on a 12-year cycle, and there may be differences in the accuracy of biochemical indices and MGUS-related indices in the population, which may affect our study results. Second, some factors that may affect nutritional status indices were not included in the model because they could not be obtained in our study population, such as gastrointestinal diseases. Finally, our study is based on a cross-sectional survey of the NHANES study and cannot make causal inferences. Therefore, our study cannot clarify whether the correlations are causal or not. Alternatively, our correlation results may also be a manifestation of chronic inflammation and monoclonal cell renewal associated with MGUS patients. Therefore, more studies and data are needed to explore the relationship between immune nutrition status and MGUS disease.

## 5. Conclusions

In summary, we found that poorer nutritional status, as assessed by PNI and CONUT, was positively associated with the risk of MGUS in middle-aged and elderly people over 50 years old by exploring the relationship between nutritional status-related indices and MGUS. This means that we may be able to focus on screening older adults with poor nutritional status for MGUS and include them in clinical management at early stage. Additionally, improving the overall nutritional status of the population may decrease the risk of developing MGUS. However, further larger prospective studies are needed to explore whether there is a causal relationship between nutritional status and MGUS risks.

## Figures and Tables

**Table 1:** Details of the nutritional indices utilized in the study.

Score	Abbreviation	Calculation Formula	Reference
Body Mass Index	BMI	weight(kg)/(height(m))^2^	
Prognostic Nutritional Index	PNI	(10 × serum albumin (g/dL)) + (0.005 × lymphocytes/μL)	[10]
Controlling Nutritional Status	CONUT	Serum albumin score + total lymphocyte count score + total cholesterol score. For serum albumin levels >3.5, between 3.0 and 3.49, between 2.5 and 2.99 and <2.5 g/dL, 0, 2, 4, and 6 points were assigned, respectively. For serum total cholesterol levels >180, between 140 and 179, between 100 and 139 and <100 mg/dL, 0, 1, 2, and 3 points were assigned, respectively. For serum total lymphocyte count >1600, between 1200 and 1599, between 800 and 1199 and <800/mm^3^, 0, 1, 2, and 3 points were assigned, respectively. Their sum ranges from <3 to ≥3.	[11]
Geriatric Nutritional Risk Index	GNRI	(1.489 × serum albumin (g/L) + 41.7 × (present weight/ideal body weight) ideal body weight = height(cm)—100—(height(cm)—150)/(4(male), 2.5(female))	[12]

**Table 2:** The baseline characteristics of all participants.

Variables	Non-MGUS	MGUS	*p*-Values
	(*n* = 9254)	(*n* = 266)	
Age—yrs, median	63.63 (63.30, 64.16)	68.83 (67.16, 70.50)	<0.001 ***^,b^
Sex			0.08
Female	53.68	46.44	
Male	46.32	53.56	
Dairy intake—kcal, median (IQR)	1784.79 (1336.00, 2348.00)	1730.76 (1277.00, 2315.00)	0.32 ^a^
Laboratory tests			
Albumin—g/L	41.97 (41.75, 42.18)	40.36 (39.62, 41.11)	<0.001 ***^,b^
Total cholesterol—mg/dL	216.84 (215.28, 218.40)	208.65 (200.98, 216.32)	0.04 *^,b^
Triglycerides—mmol/L	1.77 (1.73, 1.81)	1.81 (1.65, 1.97)	0.63 ^b^
Lymphocyte—×10^9^/L	2.13 (2.09, 2.16)	2.10 (1.95, 2.25)	0.75 ^b^
BMI—kg/m^2^, median (IQR)	27.14 (24.10, 30.88)	27.90 (24.70, 31.47)	0.39 ^a^
PNI—point, median (IQR)	52.00 (49.00, 55.50)	50.50 (46.50, 53.75)	<0.001 ***^,a^
GNRI—point, median (IQR)	114.28 (107.85, 121.91)	113.35 (104.88, 120.46)	0.048 *^,a^
BMI—kg/m^2^			0.24
normal weight	32.00	26.34	
overweight	38.47	41.51	
obese	29.52	32.14	
CONUT strata			<0.001 ***
<3	90.72	81.73	
≥3	9.28	18.27	
Race			0.20
Whites	82.20	79.92	
Blacks	7.68	11.04	
Others	10.13	9.05	
Education status			0.29
<high school	17.78	20.72	
≥high school	82.22	79.28	
Smoking			0.30
Never smoked	43.42	42.31	
Former smoker	38.80	43.77	
Current smoker	17.78	13.92	
Drinking			0.94
No	26.88	26.60	
Yes	73.12	73.40	
Poverty			0.01 *
≤1.35	18.46	14.11	
1.36–3	28.97	38.94	
>3	52.56	46.95	

*p*-values show the difference between participants with or without MGUS resulting from *t*-tests or Mann–Whitney U tests for continuous, and chi-square tests for categorical variables. a. Results from Mann–Whitney U test. b. Results from *t*-test. * Statistically significant at *p* < 0.05. *** Statistically significant at *p* < 0.001.

**Table 3:** Logistic regression analysis on the association between the nutritional indices and MGUS.

Nutritional Indices					Model 1 ^a^		Model 2 ^b^		Model 3 ^c^	
	Cases (n = 266)	Non-Cases (n = 9254)	Unadjusted or (95% CI)	*p*-Values	Multivariable Adjusted or (95% CI)	*p*-Values	Multivariable Adjusted or (95% CI)	*p*-Values	Multivariable Adjusted or (95% CI)	*p*-Values
**BMI**										
normal weight	80 (30.07%)	2878 (31.10%)	[Ref]		[Ref]		[Ref]		[Ref]	
overweight	109 (40.98%)	3699 (39.97%)	1.311 (0.900–1.910)	0.155	1.331 (0.918–1.929)	0.128	1.322 (0.911–1.918)	0.138	1.327 (0.897–1.961)	0.152
obese	77 (28.95%)	2677 (28.93%)	1.323 (0.887–1.971)	0.165	1.567 (1.051–2.335)	0.028 *	1.547 (1.024–2.336)	0.039	1.553 (1.023–2.358)	0.039 *
*p* for trend			0.155		0.026 *		0.034 *		0.036 *	
**PNI**										
Q1	112 (42.11%)	2519 (27.22%)	[Ref]		[Ref]		[Ref]		[Ref]	
Q2	67 (25.19%)	2365 (25.56%)	0.556 (0.362–0.855)	0.009 **	0.613 (0.391–0.960)	0.033 *	0.623 (0.397–0.979)	0.041 *	0.623 (0.396–0.979)	0.04 *
Q3	46 (17.29%)	2090 (22.58%)	0.511 (0.335–0.780)	0.003 **	0.586 (0.379–0.906)	0.017 *	0.602 (0.388–0.934)	0.025 *	0.600 (0.386–0.934)	0.025 *
Q4	41 (15.41%)	2280 (24.64%)	0.407 (0.236–0.703)	0.002 **	0.498 (0.286–0.867)	0.015 *	0.510 (0.289–0.902)	0.022 *	0.509 (0.290–0.896)	0.02 *
*p* for trend			<0.001 ***		0.005 **		0.008 **		0.008 **	
**GNRI**										
Q1	88 (33.08%)	2292 (24.77%)	[Ref]		[Ref]		[Ref]		[Ref]	
Q2	59 (22.19%)	2321 (25.08%)	0.611 (0.395–0.945)	0.028 *	0.656 (0.429–1.004)	0.052	0.663 (0.432–1.018)	0.06	0.660 (0.424–1.026)	0.064
Q3	63 (23.68%)	2317 (25.04%)	0.845 (0.574–1.244)	0.386	0.947 (0.648–1.382)	0.771	0.951 (0.646–1.400)	0.795	0.942 (0.639–1.388)	0.756
Q4	56 (21.05%)	2324 (25.11%)	0.577 (0.358–0.930)	0.025 *	0.742 (0.455–1.211)	0.226	0.746 (0.452–1.232)	0.245	0.737 (0.443–1.227)	0.233
*p* for trend			0.043 *		0.378		0.409		0.377	
**CONUT**										
<3	221 (83.08%)	8244 (89.09%)	[Ref]		[Ref]		[Ref]		[Ref]	
≥3	45 (16.92%)	1010 (10.01%)	2.186 (1.556, 3.073)	<0.001 ***	1.814 (1.274, 2.582)	0.001 ***	1.799 (1.264, 2.561)	0.002 **	1.805 (1.271, 2.564)	0.002 **

a. Multivariable logistic regression analysis of nutritional status indicator adjusted for age and sex. b. Multivariable logistic regression analysis of nutritional status indicator adjusted for age, sex, ethnicity, income and education level. c. Multivariable logistic regression analysis of nutritional status indicator adjusted for age, sex, ethnicity, income, education level, smoking status, drinking status and total daily energy intake. * Statistically significant at *p* < 0.05. ** Statistically significant at *p* < 0.01. *** Statistically significant at *p* < 0.001.

## Data Availability

The data used in this manuscript are publicly available at the NHANES website: https://wwwn.cdc.gov/nchs/nhanes/ (accessed on 10 July 2023).

## Supplementary Materials

The following supporting information can be downloaded at: https://www.mdpi.com/article/10.3390/nu15194210/s1. Figure S1: Association of BMI, GNRI score and PNI scores with risk of MGUS in individuals from NHANES Study. Table S1: Subgroup analysis of association between BMI and MGUS, stratified by age, sex, education level, ethnicity, PIR, smoking and drinking status. Table S2: Subgroup analysis of association between PNI and MGUS, stratified by age, sex, education level, ethnicity, PIR, smoking and drinking status. Table S3: Subgroup analysis of association between GNRI and MGUS, stratified by age, sex, education level, ethnicity, PIR, smoking and drinking status.

## Abbreviations

MGUS, monoclonal gammopathy of undetermined significance; MM, multiple myeloma; NHANES, National Health and Nutrition Examination Survey; CONUT, controlling nutritional status; PNI, prognostic nutritional index; GNRI, geriatric nutritional risk index; BMI, body mass index; PIR, poverty income ratio; MEC, mobile examination center; IQR, interquartile ranges; RCS, restricted cubic spline; HDL-C, high-density lipoprotein cholesterol.

## References

[1] Kyle R.A., Therneau T.M., Rajkumar S.V., Larson D.R., Plevak M.F., Offord J.R., Dispenzieri A., Katzmann J.A., Melton L.J. (2006). Prevalence of Monoclonal Gammopathy of Undetermined Significance. N. Engl. J. Med..

[2] Atkin C., Reddy-Kolanu V., Drayson M.T., Sapey E., Richter A.G. (2020). The Prevalence and Significance of Monoclonal Gammopathy of Undetermined Significance in Acute Medical Admissions. Br. J. Haematol..

[3] Landgren O., Kyle R.A., Pfeiffer R.M., Katzmann J.A., Caporaso N.E., Hayes R.B., Dispenzieri A., Kumar S., Clark R.J., Baris D. (2009). Monoclonal Gammopathy of Undetermined Significance (MGUS) Consistently Precedes Multiple Myeloma: A Prospective Study. Blood.

[4] Landgren O., Graubard B.I., Katzmann J.A., Kyle R.A., Ahmadizadeh I., Clark R., Kumar S.K., Dispenzieri A., Greenberg A.J., Therneau T.M. (2014). Racial Disparities in the Prevalence of Monoclonal Gammopathies: A Population-Based Study of 12,482 Persons from the National Health and Nutritional Examination Survey. Leukemia.

[5] Waxman A.J., Mink P.J., Devesa S.S., Anderson W.F., Weiss B.M., Kristinsson S.Y., McGlynn K.A., Landgren O. (2010). Racial Disparities in Incidence and Outcome in Multiple Myeloma: A Population-Based Study. Blood.

[6] Recalde M., Davila-Batista V., Díaz Y., Leitzmann M., Romieu I., Freisling H., Duarte-Salles T. (2021). Body Mass Index and Waist Circumference in Relation to the Risk of 26 Types of Cancer: A Prospective Cohort Study of 3.5 Million Adults in Spain. BMC Med..

[7] Hofmann J.N., Moore S.C., Lim U., Park Y., Baris D., Hollenbeck A.R., Matthews C.E., Gibson T.M., Hartge P., Purdue M.P. (2013). Body Mass Index and Physical Activity at Different Ages and Risk of Multiple Myeloma in the NIH-AARP Diet and Health Study. Am. J. Epidemiol..

[8] Carson K.R., Bates M.L., Tomasson M.H. (2014). The Skinny on Obesity and Plasma Cell Myeloma: A Review of the Literature. Bone Marrow Transpl..

[9] Kleinstern G., Larson D.R., Allmer C., Norman A.D., Muntifering G., Sinnwell J., Visram A., Rajkumar V., Dispenzieri A., Kyle R.A. (2022). Body Mass Index Associated with Monoclonal Gammopathy of Undetermined Significance (MGUS) Progression in Olmsted County, Minnesota. Blood Cancer J..

[10] Ignacio de Ulíbarri J., González-Madroño A., de Villar N.G.P., González P., González B., Mancha A., Rodríguez F., Fernández G. (2005). CONUT: A Tool for Controlling Nutritional Status. First Validation in a Hospital Population. Nutr. Hosp..

[11] Buzby G.P., Mullen J.L., Matthews D.C., Hobbs C.L., Rosato E.F. (1980). Prognostic Nutritional Index in Gastrointestinal Surgery. Am. J. Surg..

[12] Bouillanne O., Morineau G., Dupont C., Coulombel I., Vincent J.-P., Nicolis I., Benazeth S., Cynober L., Aussel C. (2005). Geriatric Nutritional Risk Index: A New Index for Evaluating at-Risk Elderly Medical Patients. Am. J. Clin. Nutr..

[13] Arero G., Arero A.G., Mohammed S.H., Vasheghani-Farahani A. (2022). Prognostic Potential of the Controlling Nutritional Status (CONUT) Score in Predicting All-Cause Mortality and Major Adverse Cardiovascular Events in Patients with Coronary Artery Disease: A Meta-Analysis. Front. Nutr..

[14] Chen M.-Y., Wen J.-X., Lu M.-T., Jian X.-Y., Wan X.-L., Xu Z.-W., Liang J.-Q., Wu J.-D. (2022). Association Between Prognostic Nutritional Index and Prognosis in Patients with Heart Failure: A Meta-Analysis. Front. Cardiovasc. Med..

[15] Ahn S.S., Yoo J., Jung S.M., Song J.J., Park Y.-B., Lee S.-W. (2019). Comparison of the Clinical Implications among Five Different Nutritional Indices in Patients with Lupus Nephritis. Nutrients.

[16] Correa-Rodríguez M., Pocovi-Gerardino G., Callejas-Rubio J.-L., Fernández R.R., Martín-Amada M., Cruz-Caparros M.-G., Ortego-Centeno N., Rueda-Medina B. (2019). The Prognostic Nutritional Index and Nutritional Risk Index Are Associated with Disease Activity in Patients with Systemic Lupus Erythematosus. Nutrients.

[17] Kim H., Shin D.-M., Lee J.-H., Cho E.-S., Lee H.S., Shin S.-J., Park E.J., Baik S.H., Lee K.Y., Kang J. (2023). Combining Prognostic Nutritional Index (PNI) and Controlling Nutritional Status (CONUT) Score as a Valuable Prognostic Factor for Overall Survival in Patients with Stage I-III Colorectal Cancer. Front. Oncol..

[18] Ioannidis J.P.A. (2018). The Challenge of Reforming Nutritional Epidemiologic Research. JAMA.

[19] Key T.J., Bradbury K.E., Perez-Cornago A., Sinha R., Tsilidis K.K., Tsugane S. (2020). Diet, Nutrition, and Cancer Risk: What Do We Know and What Is the Way Forward?. BMJ.

[20] Ezzati T.M., Massey J.T., Waksberg J., Chu A., Maurer K.R. (1992). Sample Design: Third National Health and Nutrition Examination Survey.

[21] Curtin L.R., Mohadjer L.K., Dohrmann S.M., Montaquila J.M., Kruszan-Moran D., Mirel L.B., Carroll M.D., Hirsch R., Schober S., Johnson C.L. (2012). The National Health and Nutrition Examination Survey: Sample Design, 1999–2006.

[22] Zhao X.-H., Shen W.-B., Wang D., Wang H.-S., Song C.-Y., Deng W.-Z. (2023). The Prognosis Value of CONUT and SIS Score for Recurrent or Metastatic Esophageal Squamous Cell Carcinoma Patients Treated with Second-Line Immunotherapy. Front. Oncol..

[23] Li Y., Zhang C., Ji R., Lu H., Zhang W., Li L.-L., Liu R., Qian H., He A. (2020). Prognostic Significance of the Controlling Nutritional Status (CONUT) Score in Epithelial Ovarian Cancer. Int. J. Gynecol. Cancer.

[24] Zhang G., Zhang Y., He F., Wu H., Wang C., Fu C. (2021). Preoperative Controlling Nutritional Status (CONUT) Score Is a Prognostic Factor for Early-Stage Cervical Cancer Patients with High-Risk Factors. Gynecol. Oncol..

[25] Toyokawa T., Kubo N., Tamura T., Sakurai K., Amano R., Tanaka H., Muguruma K., Yashiro M., Hirakawa K., Ohira M. (2016). The Pretreatment Controlling Nutritional Status (CONUT) Score Is an Independent Prognostic Factor in Patients with Resectable Thoracic Esophageal Squamous Cell Carcinoma: Results from a Retrospective Study. BMC Cancer.

[26] Harrell F.E. (2015). Regression Modeling Strategies.

[27] Landgren O., Rajkumar S.V., Pfeiffer R.M., Kyle R.A., Katzmann J.A., Dispenzieri A., Cai Q., Goldin L.R., Caporaso N.E., Fraumeni J.F. (2010). Obesity Is Associated with an Increased Risk of Monoclonal Gammopathy of Undetermined Significance among Black and White Women. Blood.

[28] Britton J.A., Khan A.E., Rohrmann S., Becker N., Linseisen J., Nieters A., Kaaks R., Tjønneland A., Halkjaer J., Severinsen M.T. (2008). Anthropometric Characteristics and Non-Hodgkin’s Lymphoma and Multiple Myeloma Risk in the European Prospective Investigation into Cancer and Nutrition (EPIC). Haematologica.

[29] Blair C.K., Cerhan J.R., Folsom A.R., Ross J.A. (2005). Anthropometric Characteristics and Risk of Multiple Myeloma. Epidemiology.

[30] Renehan A.G., Tyson M., Egger M., Heller R.F., Zwahlen M. (2008). Body-Mass Index and Incidence of Cancer: A Systematic Review and Meta-Analysis of Prospective Observational Studies. Lancet.

[31] Lin J.-X., Lin L.-Z., Tang Y.-H., Wang J.-B., Lu J., Chen Q.-Y., Cao L.-L., Lin M., Tu R.-H., Huang C.-M. (2020). Which Nutritional Scoring System Is More Suitable for Evaluating the Short- or Long-Term Prognosis of Patients with Gastric Cancer Who Underwent Radical Gastrectomy?. J. Gastrointest. Surg..

[32] Mao Y.-S., Hao S.-J., Zou C.-F., Xie Z.-B., Fu D.-L. (2020). Controlling Nutritional Status Score Is Superior to Prognostic Nutritional Index Score in Predicting Survival and Complications in Pancreatic Ductal Adenocarcinoma: A Chinese Propensity Score Matching Study. Br. J. Nutr..

[33] Yoon J.-P., Nam J.-S., Abidin M.F.B.Z., Kim S.-O., Lee E.-H., Choi I.-C., Chin J.-H. (2021). Comparison of Preoperative Nutritional Indexes for Outcomes after Primary Esophageal Surgery for Esophageal Squamous Cell Carcinoma. Nutrients.

[34] Matsukawa T., Suto K., Kanaya M., Izumiyama K., Minauchi K., Yoshida S., Oda H., Miyagishima T., Mori A., Ota S. (2020). Validation and Comparison of Prognostic Values of GNRI, PNI, and CONUT in Newly Diagnosed Diffuse Large B Cell Lymphoma. Ann. Hematol..

[35] Yang Z., Zheng Y., Wu Z., Wen Y., Wang G., Chen S., Tan F., Li J., Wu S., Dai M. (2021). Association between Pre-Diagnostic Serum Albumin and Cancer Risk: Results from a Prospective Population-Based Study. Cancer Med..

[36] He M.-M., Fang Z., Hang D., Wang F., Polychronidis G., Wang L., Lo C.-H., Wang K., Zhong R., Knudsen M.D. (2021). Circulating Liver Function Markers and Colorectal Cancer Risk: A Prospective Cohort Study in the UK Biobank. Int. J. Cancer.

[37] Kühn T., Sookthai D., Graf M.E., Schübel R., Freisling H., Johnson T., Katzke V., Kaaks R. (2017). Albumin, Bilirubin, Uric Acid and Cancer Risk: Results from a Prospective Population-Based Study. Br. J. Cancer.

[38] Greipp P.R., San Miguel J., Durie B.G.M., Crowley J.J., Barlogie B., Bladé J., Boccadoro M., Child J.A., Avet-Loiseau H., Kyle R.A. (2005). International Staging System for Multiple Myeloma. J. Clin. Oncol..

[39] Palumbo A., Avet-Loiseau H., Oliva S., Lokhorst H.M., Goldschmidt H., Rosinol L., Richardson P., Caltagirone S., Lahuerta J.J., Facon T. (2015). Revised International Staging System for Multiple Myeloma: A Report from International Myeloma Working Group. J. Clin. Oncol..

[40] D’Agostino M., Cairns D.A., Lahuerta J.J., Wester R., Bertsch U., Waage A., Zamagni E., Mateos M.-V., Dall’Olio D., van de Donk N.W.C.J. (2022). Second Revision of the International Staging System (R2-ISS) for Overall Survival in Multiple Myeloma: A European Myeloma Network (EMN) Report Within the HARMONY Project. J. Clin. Oncol..

[41] Kyle R.A., Therneau T.M., Rajkumar S.V., Remstein E.D., Offord J.R., Larson D.R., Plevak M.F., Melton L.J. (2003). Long-Term Follow-up of IgM Monoclonal Gammopathy of Undetermined Significance. Blood.

[42] Kadakia K.C., Symanowski J.T., Aktas A., Szafranski M.L., Salo J.C., Meadors P.L., Walsh D. (2022). Malnutrition Risk at Solid Tumor Diagnosis: The Malnutrition Screening Tool in a Large US Cancer Institute. Support Care Cancer.

[43] Halliwell B. (1988). Albumin--an Important Extracellular Antioxidant?. Biochem. Pharmacol..

[44] de Mutsert R., Grootendorst D.C., Indemans F., Boeschoten E.W., Krediet R.T., Dekker F.W. (2009). Netherlands Cooperative Study on the Adequacy of Dialysis-II Study Group Association between Serum Albumin and Mortality in Dialysis Patients Is Partly Explained by Inflammation, and Not by Malnutrition. J. Ren. Nutr..

[45] Liu Y., Tian S., Ning B., Huang T., Li Y., Wei Y. (2022). Stress and Cancer: The Mechanisms of Immune Dysregulation and Management. Front. Immunol..

[46] Tienhaara A., Pelliniemi T.T. (1994). Peripheral Blood Lymphocyte Subsets in Multiple Myeloma and Monoclonal Gammopathy of Undetermined Significance. Clin. Lab. Haematol..

[47] Prabhala R.H., Neri P., Bae J.E., Tassone P., Shammas M.A., Allam C.K., Daley J.F., Chauhan D., Blanchard E., Thatte H.S. (2006). Dysfunctional T Regulatory Cells in Multiple Myeloma. Blood.

[48] Pedersen K.M., Çolak Y., Bojesen S.E., Nordestgaard B.G. (2020). Low High-Density Lipoprotein and Increased Risk of Several Cancers: 2 Population-Based Cohort Studies Including 116,728 Individuals. J. Hematol. Oncol..

[49] Choi T., Choi I.Y., Han K., Jeong S.-M., Yoo J.E., Rhee S.Y., Park Y.-G., Shin D.W. (2021). Lipid Level, Lipid Variability, and Risk of Multiple Myeloma: A Nationwide Population-Based Study of 3,527,776 Subjects. Cancers.

[50] Landgren O., Graubard B.I., Kumar S., Kyle R.A., Katzmann J.A., Murata K., Costello R., Dispenzieri A., Caporaso N., Mailankody S. (2017). Prevalence of Myeloma Precursor State Monoclonal Gammopathy of Undetermined Significance in 12372 Individuals 10-49 Years Old: A Population-Based Study from the National Health and Nutrition Examination Survey. Blood Cancer J..

[51] Wang Y.-Z., Wu Q.-J., Zhu J., Wu L. (2015). Fish Consumption and Risk of Myeloma: A Meta-Analysis of Epidemiological Studies. Cancer Causes Control.

[52] Thordardottir M., Lindqvist E.K., Lund S.H., Costello R., Burton D., Steingrimsdottir L., Korde N., Mailankody S., Eiriksdottir G., Launer L.J. (2018). Dietary Intake Is Associated with Risk of Multiple Myeloma and Its Precursor Disease. PLoS ONE.

[53] Hosgood H.D., Baris D., Zahm S.H., Zheng T., Cross A.J. (2007). Diet and Risk of Multiple Myeloma in Connecticut Women. Cancer Causes Control.

