# Supplementary material for: Nutritional Status Indices and Monoclonal Gammopathy of Undetermined Significance Risk in the Elderly Population: Findings from the National Health and Nutrition Examination Survey
Source: Nutrients. 2023 Sep 29;15(19):4210. doi: 10.3390/nu15194210 (PMC10574740; doi:10.3390/nu15194210)
Supplement: Supplementary file 1 [file nutrients-15-04210-s001.zip › nutrients-2605151-supplementary.pdf]

Table S1. Subgroup analysis of association between BMI and MGUS, stratified by age, sex, education level, ethnicity, PIR, smoking and drinking status.

Character	Normal weight	Overweight	*p*-Values	Obese	*p*-Values	*p* for trend	*p* for interaction
Age—yrs							0.584
<70	[Ref]	1.300 (0.694,2.433)	0.405	1.655 (0.923,2.970)	0.089	0.076	
≥70	[Ref]	1.414 (0.920,2.172)	0.112	1.257 (0.721,2.192)	0.412	0.309	
Sex							0.204
Female	[Ref]	0.932 (0.528,1.645)	0.805	1.053 (0.600,1.848)	0.855	0.866	
Male	[Ref]	1.741 (1.040,2.912)	0.035*	1.744 (0.995,3.057)	0.052	0.044*	
Education status							0.612
＜high school	[Ref]	1.007 (0.425,2.386)	0.987	0.902 (0.383,2.123)	0.809	0.803	
≥high school	[Ref]	1.399 (0.949,2.062)	0.089	1.451 (0.924,2.278)	0.104	0.098	
Race							0.836
Whites	[Ref]	1.349 (0.875,2.079)	0.171	1.291 (0.793,2.104)	0.297	0.278	
Blacks	[Ref]	1.050 (0.507,2.173)	0.893	0.977 (0.478,1.997)	0.947	0.928	
Others	[Ref]	1.240 (0.397,3.870)	0.706	1.903 (0.675,5.367)	0.218	0.238	
Poverty							0.053
≤1.35	[Ref]	2.576 (1.285,5.164)	0.009**	1.787 (0.746,4.281)	0.188	0.219	
1.36-3	[Ref]	1.012 (0.526,1.944)	0.972	0.646 (0.333,1.254)	0.191	0.195	
>3	[Ref]	1.376 (0.666,2.842)	0.381	2.130 (1.084,4.186)	0.029*	0.022*	
Drinking							0.240
No	[Ref]	0.951 (0.451,2.007)	0.893	0.757 (0.317,1.807)	0.523	0.517	
Yes	[Ref]	1.489 (0.967,2.293)	0.070	1.645 (1.022,2.646)	0.041*	0.033*	
Smoking							0.049*
Never smoked	[Ref]	0.725 (0.388,1.355)	0.307	0.771 (0.423,1.405)	0.388	0.387	
Former smoker	[Ref]	1.965 (0.996,3.878)	0.051	2.301 (1.280,4.138)	0.006**	0.003**	
Current smoker	[Ref]	2.736 (0.893,8.378)	0.077	1.314 (0.379,4.554)	0.661	0.350	

PIR: poverty income ratio

*Statistically significant at $p < 0.05$.

**Statistically significant at $p < 0.01$.

***Statistically significant at $p < 0.001$.

Table S2. Subgroup analysis of association between PNI and MGUS, stratified by age, sex, education level, ethnicity, PIR, smoking and drinking status.

Character	Q1	Q2	*p*-Values	Q3	*p*-Values	Q4	*p*-Values	*p* for trend	*p* for interaction
Age—yrs									0.005**
<70	[Ref]	0.321 (0.161,0.640)	0.002**	0.571 (0.304,1.073)	0.081	0.498 (0.246,1.006)	0.052	0.113	
≥70	[Ref]	0.995 (0.616,1.605)	0.982	0.517 (0.288,0.930)	0.028*	0.349 (0.180,0.676)	0.002**	<0.001***	
Sex									0.175
Female	[Ref]	0.723 (0.412,1.269)	0.252	0.604 (0.312,1.171)	0.132	0.647 (0.282,1.484)	0.296	0.248	
Male	[Ref]	0.416 (0.229,0.756)	0.005**	0.412 (0.256,0.665)	<0.001***	0.245 (0.121,0.495)	<0.001***	<0.0001***	
Education status									0.055
＜high school	[Ref]	0.967 (0.433,2.161)	0.934	0.367 (0.128,1.049)	0.061	0.219 (0.074,0.645)	0.007**	0.001***	
≥high school	[Ref]	0.457 (0.275,0.758)	0.003**	0.546 (0.347,0.860)	0.010*	0.462 (0.256,0.834)	0.012*	0.005**	
Race									0.871
Whites	[Ref]	0.513 (0.307,0.856)	0.012*	0.530 (0.326,0.862)	0.012*	0.425 (0.224,0.807)	0.010*	0.004**	
Blacks	[Ref]	0.822 (0.383,1.763)	0.605	0.409 (0.139,1.202)	0.101	0.465 (0.179,1.206)	0.112	0.059	
Others	[Ref]	0.699 (0.190,2.570)	0.583	0.486 (0.159,1.484)	0.200	0.273 (0.068,1.101)	0.067	0.037*	
Poverty									0.162
≤1.35	[Ref]	1.647 (0.685,3.957)	0.258	1.122 (0.381,3.305)	0.831	0.744 (0.205,2.697)	0.646	0.476	
1.36-3	[Ref]	0.708 (0.380,1.316)	0.268	0.552 (0.281,1.084)	0.083	0.347 (0.157,0.765)	0.010*	0.002**	
>3	[Ref]	0.329 (0.164,0.662)	0.002**	0.406 (0.206,0.803)	0.011*	0.407 (0.211,0.785)	0.008**	0.006**	
Drinking									0.771
No	[Ref]	0.692 (0.272,1.760)	0.432	0.766 (0.317,1.849)	0.546	0.504 (0.213,1.193)	0.116	0.112	
Yes	[Ref]	0.513 (0.324,0.813)	0.005**	0.443 (0.270,0.729)	0.002**	0.377 (0.195,0.729)	0.005**	0.001**	
Smoking									0.744
Never smoked	[Ref]	0.649 (0.340,1.237)	0.184	0.439 (0.244,0.791)	0.007**	0.476 (0.229,0.992)	0.048*	0.016*	
Former smoker	[Ref]	0.565 (0.320,0.999)	0.050	0.627 (0.353,1.111)	0.108	0.361 (0.175,0.745)	0.007**	0.003**	
Current smoker	[Ref]	0.271 (0.087,0.843)	0.025*	0.372 (0.117,1.185)	0.093	0.359 (0.111,1.166)	0.087	0.180	

PIR: poverty income ratio

*Statistically significant at p < 0.05.

**Statistically significant at p < 0.01.

***Statistically significant at p < 0.001.

Table S3. Subgroup analysis of association between GNRI and MGUS, stratified by age, sex, education level, ethnicity, PIR, smoking and drinking status.

Table S3. Subgroup analysis of GNRI.

Character	Q1	Q2	*p*-Values	Q3	*p*-Values	Q4	*p*-Values	*p* for trend	*p* for interaction
Age—yrs									0.915
<70	[Ref]	0.579 (0.263,1.276)	0.171	0.963 (0.473,1.959)	0.916	0.692 (0.361,1.328)	0.262	0.482	
≥70	[Ref]	0.737 (0.424,1.280)	0.272	0.903 (0.549,1.484)	0.680	0.653 (0.349,1.220)	0.177	0.221	
Sex									0.640
Female	[Ref]	0.481 (0.256,0.903)	0.024*	0.638 (0.372,1.092)	0.099	0.552 (0.286,1.066)	0.076	0.104	
Male	[Ref]	0.735 (0.405,1.336)	0.306	1.008 (0.549,1.848)	0.980	0.599 (0.323,1.111)	0.102	0.176	
Education status									0.444
＜high school	[Ref]	0.386 (0.178,0.841)	0.018*	0.541 (0.256,1.142)	0.105	0.401 (0.170,0.947)	0.038*	0.067	
≥high school	[Ref]	0.689 (0.423,1.123)	0.132	0.952 (0.616,1.470)	0.821	0.636 (0.362,1.115)	0.112	0.179	
Race									0.564
Whites	[Ref]	0.513 (0.301,0.874)	0.015*	0.831 (0.528,1.308)	0.415	0.523 (0.290,0.944)	0.032*	0.069	
Blacks	[Ref]	1.075 (0.524,2.205)	0.839	1.058 (0.446,2.510)	0.896	0.751 (0.323,1.748)	0.497	0.472	
Others	[Ref]	1.427 (0.396,5.138)	0.579	0.925 (0.183,4.661)	0.923	1.086 (0.426,2.772)	0.859	0.873	
Poverty									0.624
≤1.35	[Ref]	0.828 (0.361,1.897)	0.649	1.469 (0.647,3.335)	0.350	0.646 (0.244,1.710)	0.371	0.524	
1.36-3	[Ref]	0.497 (0.244,1.012)	0.054	0.681 (0.340,1.364)	0.272	0.380 (0.185,0.782)	0.010*	0.023*	
>3	[Ref]	0.691 (0.354,1.347)	0.271	0.868 (0.414,1.822)	0.702	0.783 (0.388,1.581)	0.487	0.626	
Drinking									0.372
No	[Ref]	0.352 (0.144,0.860)	0.023*	0.551 (0.220,1.377)	0.197	0.371 (0.146,0.945)	0.038*	0.081*	
Yes	[Ref]	0.741 (0.452,1.215)	0.229	0.995 (0.637,1.553)	0.981	0.685 (0.421,1.113)	0.123	0.190	
Smoking									0.337
Never smoked	[Ref]	0.338 (0.174,0.655)	0.002**	0.536 (0.293,0.981)	0.043*	0.470 (0.249,0.888)	0.021*	0.055	
Former smoker	[Ref]	0.810 (0.386,1.698)	0.569	1.181 (0.678,2.059)	0.549	0.686 (0.350,1.344)	0.265	0.309	
Current smoker	[Ref]	1.191 (0.335,4.234)	0.783	0.989 (0.306,3.197)	0.985	0.480 (0.140,1.654)	0.239	0.261	

PIR: poverty income ratio

*Statistically significant at $p < 0.05$.

**Statistically significant at $p < 0.01$.

***Statistically significant at $p < 0.001$.

Supplementary Figure S1. Association of BMI, GNRI score and PNI scores with risk of MGUS in individuals from NHANES Study.

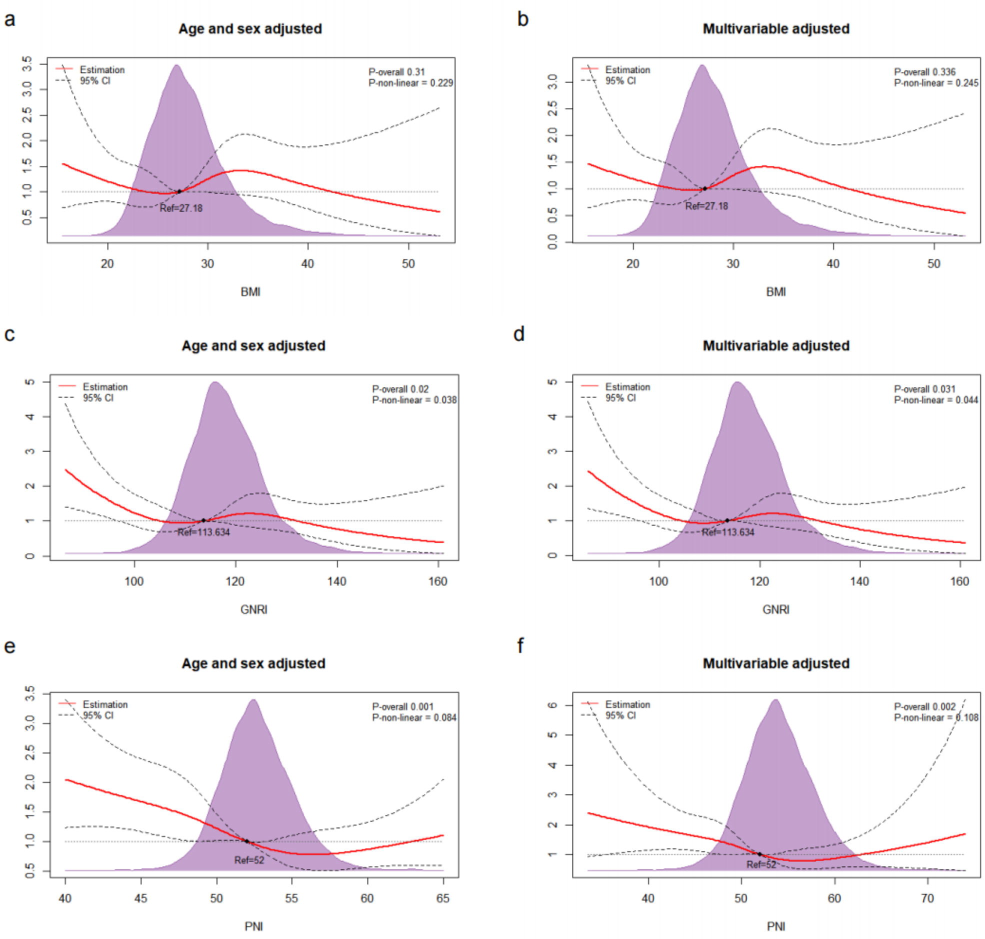

Odds ratios and 95% confidence intervals (CIs) were obtained from logistic regression with restricted cubic splines. Multivariable adjustment included age, sex, ethnicity, income, education level, smoking status, drinking status and total daily energy intake. The median values of each nutritional indices were chosen as reference. The red line represents the odds ratio and the dotted lines 95% CIs. Areas of purple represent the distribution of levels of each nutritional index. P-overall is calculated each parameter as a continuous variable in logistic model. P-non-linear was calculated by Wald Chi-square test.
